# Immune Responses to Gram-Negative Bacteria in Hemolymph of the Chinese Horseshoe Crab, *Tachypleus tridentatus*

**DOI:** 10.3389/fimmu.2020.584808

**Published:** 2021-01-29

**Authors:** Wei-Feng Wang, Xiao-Yong Xie, Kang Chen, Xiu-Li Chen, Wei-Lin Zhu, Huan-Ling Wang

**Affiliations:** ^1^ Key Lab of Freshwater Animal Breeding, Key Laboratory of Agricultural Animal Genetics, Breeding and Reproduction, Ministry of Education, College of Fisheries, Huazhong Agricultural University, Wuhan, China; ^2^ Key Laboratory of South China Sea Fishery Resources Exploitation & Utilization, Ministry of Agriculture, South China Sea Fisheries Research Institute, Chinese Academy of Fishery Sciences, Guangzhou, China; ^3^ Guangxi Key Laboratory of Aquatic Genetic Breeding and Healthy Aquaculture, Guangxi Academy of Fishery Sciences, Nanning, China

**Keywords:** *Tachypleus tridentatus*, Gram-negative bacteria, innate immunity, transcriptomics, peptidomics

## Abstract

Chinese horseshoe crab, *Tachypleus tridentatus*, is an ancient marine arthropod with a long evolutionary history. As a kind of living fossil species, the pathogen defenses of horseshoe crabs entirely depend on the innate immune system. Although, there are abundant immune molecules found in the horseshoe crab hemolymph, the biological mechanisms underlying their abilities of distinguishing and defending against invading microbes are still unclear. In this study, we used high-throughput sequencing at mRNA and protein levels and bioinformatics analysis methods to systematically analyze the innate immune response to Gram-negative bacteria in hemolymph of Chinese horseshoe crab. These results showed that many genes in the complement and coagulation cascades, Toll, NF-κB, C-type lectin receptor, JAK-STAT, and MAPK signaling pathways, and antimicrobial substances were activated at 12 and 24 h post-infection, suggesting that Gram-negative bacteria could activate the hemolymph coagulation cascade and antibacterial substances release *via* the above pathways. In addition, we conjectured that Toll and NF-κB signaling pathway were most likely to participate in the immune response to Gram-negative bacteria in hemolymph of horseshoe crab through an integral signal cascade. These findings will provide a useful reference for exploring the ancient original innate immune mechanism.

## Introduction

Immunity to infectious agents is mediated by two general systems, innate and acquired immunity. Innate immunity is the first line of self-defense against infectious microbes, and is conserved within species (1). Innate immunity develops earlier than acquired immunity and has been identified in all multicellular organisms as an essential system for host defense against bacterial, fungal, and viral pathogens (2, 3). Therefore, the innate immune system is an important object of studies with regard to biological and immunological host defense reactions (4). Studies on innate immunity in animals will elucidate further the basic mechanisms to distinguish self and non-self materials (5).

Chinese horseshoe crab, *Tachypleus tridentatus*, is an ancient marine arthropod with a long evolutionary history extending back approximately 500 million years, lacking an acquired, memory-type immunity based on T-lymphocyte subsets and clonally derived immunoglobulins (2, 6). As a group of living fossil, horseshoe crabs in morphology have barely changed in the past 200 million years. Previous studies have shown that its hemolymph contains a rich repertoire of innate immune molecules, so horseshoe crab is a useful invertebrate model for exploring the innate immune mechanism (7–11). The resistance of horseshoe crabs to pathogenic invasion depends entirely on the hard carapace and the innate immune system including humoral and cellular responses (2). Similar to other invertebrates, innate immune system of horseshoe crabs includes hemolymph coagulation, phenoloxidase activation, cell agglutination, release of antibacterial substances, active oxygen formation, and phagocytosis (1, 12–14). The presence of circulating hemocytes is essential to innate immunity of horseshoe crabs. In horseshoe crabs, granular hemocytes account for 99% of all hemocytes, and are involved in the storage and release of a variety of defense molecules, including serine protease zymogens, the clottable protein coagulogen, protease inhibitors, antimicrobial peptides, and lectins (15).

Immune response in hemolymph of horseshoe crabs is triggered by pathogen-associated molecular patterns (PAMPs) present on surface of microbes, such as lipopolysaccharides (LPS) of Gram-negative bacteria, β-1,3-glucans of fungi, and peptidoglycans of Gram-positive bacteria (16). Generally, PAMPs are recognized *via* a set of pattern-recognition receptors (PRRs) that are germline-encoded receptors of the innate immune system (15). In horseshoe crabs, there are some special serine proteases in granular hemocytes, including Prochelicerase C, Prochelicerase B, and Prochelicerase G, which can directly recognize LPS and β-1,3-glucans and activate the innate immune response (15, 17, 18). However, the cell-surface receptors in horseshoe crabs have not been identified. In addition, a Toll-like protein named tToll has been identified in horseshoe crab hemocytes and shown to be expressed in multiple tissues (5, 19). Interestingly, there is no evidence to demonstrate that the tToll protein is involved in pathogens recognition. Obviously, the innate immune mechanism of horseshoe crabs is unique and complex, especially against Gram-negative bacteria containing LPS in the cell wall membrane. In previous studies, many innate immunity-related genes have been reported in Chinese horseshoe crab (13, 14, 17, 20), but the immune response mechanism based on high-throughput analysis of pathogen challenge has been rarely reported.

In the present study, we used Gram-negative *Vibrio parahaemolyticus*, a widely distributed marine pathogenic bacteria, to infect Chinese horseshoe crab by injection. Then, the comparative RNA-seq-based transcriptome combined with Label-free-based quantitative peptidome was used to analyze the expression patterns of Chinese horseshoe crab hemolymph in response to Gram-negative bacteria. This work will provide further insights into the possible immune defense mechanism in Chinese horseshoe crab hemolymph against Gram-negative bacteria.

## Materials and Methods

### Sample Collection

Artificially raised Chinese horseshoe crabs (120 ± 20 g) were obtained from aquaculture research base of South China Sea Fisheries Research Institute, Chinese Academy of Fishery Sciences (Zhanjiang, China) and maintained in the laboratory of Guangxi Academy of Fishery Science (Nanning, China) with artificial seawater at 26°C. All animals were acclimated at least 48 h under laboratory conditions.

### Bacterial Challenge and Hemolymph Collection

Gram-negative bacteria (*V. parahaemolyticus*) were cultured overnight in high salinity (3%) Luria-Bertani broth at 37°C. Bacteria were pelleted at 4,000 r/min for 5 min, washed, and resuspended in 0.9% saline to a density of 1.0×10^7^/ml. These horseshoe crabs were injected at the base of the sixth pereopod with 1.2×10^7^ CFU per kilogram of body weight (8). To explore the patterns of immune response in Chinese horseshoe crab, hemolymph (three replicates for every time point) were collected at 0, 12, 24, 36, and 72 h post infection (hpi). Samples with 0 h were considered as the control group injected with an equal volume of 0.9% saline. These samples were immediately snap-frozen in liquid nitrogen, and stored at -80°C for further analysis.

### Transcriptome Sequencing

RNA was extracted from the hemolymph of Chinese horseshoe crabs using TRIzol^®^ Reagent (Ambion, Life Technologies) according to the manufacturer’s instructions, and treated with DNase I (Invitrogen). Quality and quantity of the extracted RNA were assessed by electrophoresis in 1% agarose gels and NanoDrop 2000 spectrometer (Thermo Fisher Scientific, USA) with the threshold for A260/A280 > 1.8. Transcriptome sequencing was conducted by Shanghai Majorbio Bio-pharm Technology Co., Ltd (Shanghai, China) on the Illumina HiSeq NovaSeq 6000 (Illumina, USA) genomic sequencing platform to generate 2×150 bp paired-end reads. Eight cDNA libraries from three time points (0, 12, and 24 h) were established and sequenced with three biological repeats except the point of 12 h (two repeats). The dataset was available from the NCBI Short Read Archive (SRA) with an accession number SRP267502.

### *De Novo* Assembly and Annotation

The raw paired end reads were trimmed, and quality was controlled by SeqPrep (https://github.com/jstjohn/SeqPrep) and Sickle (https://github.com/najoshi/sickle) with default parameters. Then clean data were used to do *de novo* assembly with Trinity (http://trinityrnaseq.sourceforge.net/) (21). All these assembled transcripts were searched against NCBI protein non-redundant (NR), String, and KEGG databases using BLASTX to identify the proteins that had the highest sequence similarity with the given transcripts to retrieve their function annotations and a typical cut-off E-values less than 1.0 × 10^−5^ was set. BLAST2GO (22) program was used to get Gene Ontology (GO) annotations of unique assembled transcripts for describing biological processes, molecular functions and cellular components. Biological pathway analysis was performed using the Kyoto Encyclopedia of Genes and Genomes (KEGG, http://www.genome.jp/kegg/).

### Differential Expression Analysis and Functional Enrichment

To identify differentially expressed genes (DEGs) in two infection groups compared with the control, the expression level of each transcript was calculated according to the fragments per kilobase of exon per million mapped reads (FRKM) method. RSEM (23) was used to quantify abundances of genes. R statistical package software DESeq2 (Empirical analysis of Digital Gene Expression in R) (24) was utilized for differential expression analysis. In addition, functional enrichment analysis including GO and KEGG were performed by Goatools (25) and KOBAS (26) to identify DEGs which were significantly enriched in GO terms and biological pathways at Bonferroni-corrected *P*-value ≤0.05 compared with the whole-transcriptome background.

### Protein Extraction and Digestion

To explore the response at protein level, three individuals from the control and infection groups (24 hpi) were collected respectively for peptidome analysis. The frozen hemolymph was melted on ice. 500 μl of hemolymph was taken from each sample and placed in a new tube containing 1,500 μl of 0.4% acetonitrile, and ultrasonicated for 9 min. After standing at -20°C for 20 min and centrifuging at 14,000 r/min for 20 min, the supernatant was dried by suction filtration. Then, proteins were incubated with 50 μl of reducing agent buffer (10 mM TCEP, 8 M urea, 100 mM TEAB, pH 8.0) at 60°C for 1 h. Reduced proteins were then alkylated for 40 min at room temperature in the dark by adding iodoacetamide to a final concentration of 40 mM. The trypsin solution was added with the proportion of 1:50 (w/w) and incubated at 37°C overnight (27). After the pH of the samples was adjusted to pH = 3 by TFA, it were desalted using Sep-Pak^®^ C18 desalting column. The proteins were collected using the eluent buffer (70% ACN and 0.1% TFA), and diluted to 50% ACN using 0.2% TFA Buffer. Samples were repeatedly desalted using an Oasis^®^ MCX desalting column and eluted with an eluent buffer (70% ACN and 5% NH_4_OH). These samples were dried by suction filtration and dissolved in enzyme-free water. Proteins were quantified using the BCA protein concentration assay (28).

### High-pH RPLC Fractionation and LC-MS/MS Analysis

RPLC fractionation and LC-MS/MS analysis were completed at Shanghai Majorbio Bio-pharm Technology Co., Ltd (Shanghai, China). Samples were fractionated using high-pH reverse phase separation to increase proteomic depth (29). The peptides were resuspended with loading buffer (Ammonium hydroxide solution containing 2% acetonitrile, pH 10), separated by high-pH reversed-phase liquid chromatography (RPLC, Acquity Ultra Performance LC, Waters, USA). The gradient elution was performed on high-pH RPLC column (ACQUITY UPLC BEH C18 Column 1.7 µm, 2.1 mm X 150 mm, Waters, USA) at 200 μl/min with the gradient increased for 66 min (Phase B: Ammonium hydroxide solution containing 80% acetonitrile, pH 10; Phase A: Ammonium hydroxide solution containing 2% acetonitrile, pH 10). Twenty fractions were collected from each sample which was subsequently pooled resulting in ten total fractions per sample.

Mass spectrometry analysis was performed on a Q Exactive mass spectrometer that was coupled with Easy-nLC 1200. Each peptide sample was injected for nanoLC-MS/MS analysis. The sample was loaded onto a the C18-reversed phase column (75 μm x 25 cm, Thermo,USA) in buffer A (2% acetonitrile and 0.1% Formic acid) and separated with a linear gradient of buffer B (80% acetonitrile and 0.1% Formic acid) at a flow rate of 300 nl/min. The electrospray voltage of 1.8 kV versus the inlet of the mass spectrometer was used. Q Exactive mass spectrometer was operated in the data-dependent mode to switch automatically between MS and MS/MS acquisition. Survey full-scan MS spectra (m/z 350-1300) were acquired with a mass resolution of 70K, followed by twenty sequential high energy collisional dissociation (HCD) MS/MS scans with a resolution of 17.5K. In all cases, one microscan was recorded using dynamic exclusion of 18 s.

### Peptide Identification and Quantification

MS/MS spectra were searched using Proteome Discoverer 2.2 software against transcriptome-based protein sequences (this study). The highest score for a given peptide mass (best match to that predicted in the database) was used to identify parent proteins. The parameters for protein searching were set as follows: tryptic digestion with two missed cleavages, carbamidomethylation of cysteines as fixed modification, and oxidation of methionines and protein N-terminal acetylation as variable modifications. Peptide spectral matches were validated based on q-values at a 1% false discovery rate (FDR).

Peptide quantification was performed using Proteome Discoverer 2.2 and the fold changes in peptides between the infection and control groups were calculated. The thresholds of fold change (>1.2 or <0.83) and *P*-value <0.05 were used to identify differentially abundant peptides (DAPs). DAPs were further used to identify differentially expressed proteins (DEPs) with the cut-off value |ΔPvalue| >2 (30).

### Quantitative Real-Time PCR

Quantitative real-time PCR (qRT-PCR) was used to investigate the target gene expression patterns in different time points (0, 12, 24, 36, and 72 h) of hemolymph after infection. The primers (**Table 1**) for qRT-PCR were designed using Primer Premier 5.0 software (Premier, Canada) based on the transcript sequences. Quantitation was conducted using a CFX Connect™ Real-Time PCR detection system (Bio-Rad Laboratories, Inc, USA). The qRT-PCR mixture reaction volume was 20 µl, containing 10 µl LightCycler^®^ 480 SYBR Green I Master, 8 µl ddH_2_O, 0.5 µl of each primer (10 mM), and 1 µl cDNA template. PCR amplification was performed in triplicate, using the following conditions: an initial denaturation at 95°C for 5 min, followed by 40 cycles of 15 s at 95°C, 30 s at 60°C, and 20 s at 72°C. A dissociation stage was performed after thermo-cycling to determine target specificity. Expression levels of the tested genes were determined by Ct values and calculated by 2^-△△Ct^ (31), and 18s RNA was selected as the reference gene.

**Table 1:** Primers used for qRT-PCR in this study.

Gene names	Sequences (5’~3’)	Gene number	Tm (˚C)
Glutathione S-transferase	F: CCTTCACCTAACTCACTAACCA R: TGACGCAGATAGCCTTGTTG	TRINITY_DN1568_c0_g1	60
NF-κB p105	F: GCAAGAGTGGATTAGTGAAGTT R: TGCCCAAGTTAGTGACAGC	TRINITY_DN3661_c0_g5	60
IκB	F: GCTCGTCTAACCATTCAGTTG R: GCATGAGTATGCCATGTTGTAT	TRINITY_DN3759_c0_g1	60
Peptidase C1	F: TTCGGCTGAATAAGACAAGGTT R: CGTTCGCTATAACATTGGACAA	TRINITY_DN10879_c0_g3	60
Tachycitin	F: ATAAGCGTTGTAGTGAAGTCCT R: TTCGTTGCGGAAGGTATAGC	TRINITY_DN1090_c1_g1	60
Factor D	F: ACCCAACACTCGTCCATTATG R: ACACTCATCAATACCCGATTCT	TRINITY_DN2056_c1_g1	60
Prochelicerase B	F: CATCAGGACAGCAGACAATAGG R: CAAGACACGGAACGACAATGA	TRINITY_DN7453_c0_g1	60
Trichohyalin	F: AGATTACTGTGAGCGAAAGACT R: CTAACGGAGAAGGGAAAGATGT	TRINITY_DN1142_c0_g2	60
18s RNA	F: CGGAAGCACGAAGGAAGGA R: TCGCTCGCCGTTACTAAGG	TRINITY_DN238_c0_g1	60

### Statistical Analysis

Data from qRT-PCR were presented as the means of three biological replicates ± SD. The statistical significance was assessed by two-tailed independent t-test. *P* < 0.05 value was considered to be statistically significant difference and *P* < 0.01 value as extreme difference.

## Results

### Characteristics of Transcriptomic and Peptidomic Data

Approximately 49,988,124 to 60,096,722 clean reads were generated from eight cDNA libraries of hemolymph samples obtained from the control (0 h) and infection groups at 12 and 24 h after injection. Among the clean reads, 83.88%–86.63% were mapped to the transcripts. Based on the mapped results, the clean reads were assembled into 140,354 transcripts belonging to 114,636 predicted unigenes. A total of 238,725 spectrums and 3,515 peptide sequences were identified (FDR <1%) by combining high-throughput Label-free technology (32) with high-resolution LC-MS/MS technology. These peptides were classified into 550 specific proteins by mapping to the transcriptome dataset. These results were shown in **Table 2**.

**Table 2:** The statistics of transcriptic and proteomic data.

Types of data	Number
Raw reads	50,423,080~60,439,492
Clean reads	49,988,124~60,096,722
Transcripts	148,346
Unique genes	115,186
N50 (transcript)	2,354
N50 (unigene)	1,990
Mapped rate	83.88%~86.63%
Total Spectrum	238,725
Peptide sequences	3,515
Reliable proteins	550

### Differentially Expressed Genes and Functional Annotation

**Figure 1:**
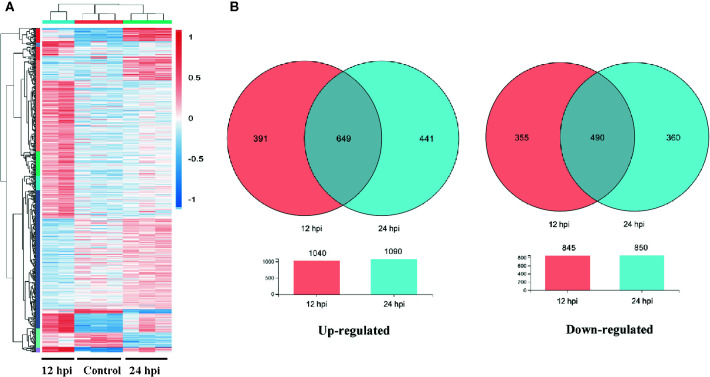
The differentially expressed gene (DEG)**** analysis at different time post infection with Gram-negative bacteria (*P* < 0.001). **(A)** A heatmap was used to classify the DEG expression patterns in all groups, the X-axis represents the time (h)**** post infection. **(B)** Venn diagrams of DEGs in 12 and 24 h groups. Left panel: up-regulated genes; right panel: down-regulated genes.

A total of 1,885 and 1,940 differentially expressed genes (DEGs) between the infection and control groups were identified at 12 and 24 hpi, respectively (*P*-value < 0.001, **Table S1**). These results were clearly visualized by clustering the samples from differential time points and constructing a Venn diagram of DEGs (**Figure 1**). The cluster analysis of all DEGs showed the differences of these samples (12 and 24 hpi) to a certain extent (**Figure 1A**). At 12 and 24 hpi, 1,040 and 1,090 genes were up-regulated, and 845 and 850 genes were down-regulated, respectively, compared with the control (**Figure 1B**, **Table S1**).

**Figure 2:**
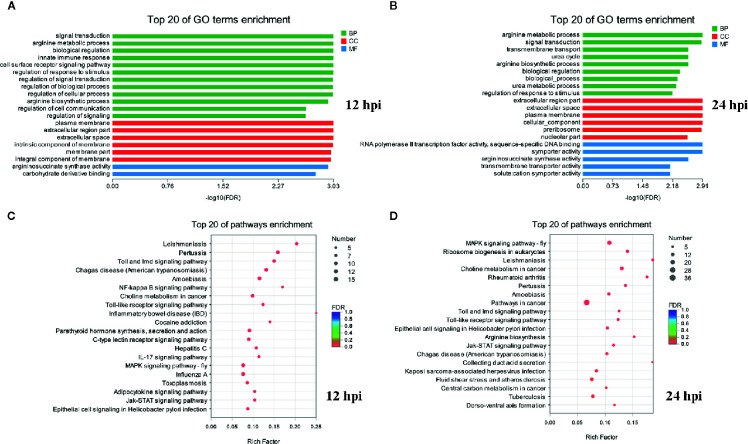
Functional enrichment analysis identified major biological processes and pathways after infection. **(A, B)** GO enrichment analysis of up-regulated genes at 12 and 24 hpi. **(C, D)** KEGG enrichment analysis of up-regulated genes at 12 and 24 hpi.

GO and KEGG pathway annotation analysis was performed to filter the biological processes and pathways for these up-regulated genes in two infection groups. The top 20 GO terms and KEGG pathways of the two groups were shown in **Figure 2**. The common enrichment GO terms in the two infection groups were signal transduction, arginine metabolic process, biological regulation, regulation of response to stimulus, plasma membrane, extracellular region part, extracellular space, arginine biosynthetic process, and argininosuccinate synthase activity (**Figures 2A, B**). In addition, some immune-related biological process, including innate immune response, immune system process, immune response, and Toll-like receptor signaling pathway, were enriched in up-regulated DEGs (**Table S2**). In the enrichment analysis of KEGG pathways, some of these pathways were related to immune responses, including Toll and Imd, Jak-STAT, NF-κB, C-type lectin receptor, and IL-17 signaling pathways. The pathways associated with pathogenic infections were significantly enriched in two infection groups, such as leishmaniasis, pertussis, inflammatory bowel disease, and epithelial cell signaling in *Helicobacter pylori* infection. Notably, MAPK signaling pathway was the most prominent pathway in the two infection groups (**Table S2**). This may reflect a common characteristic of Gram-negative bacterial infection.

GO and KEGG enrichment analysis of down-regulated genes showed that few GO terms and KEGG pathways were significantly enriched (*P* < 0.05) both in two infection groups (**Table S2**). Eight GO terms (including defense response, response to stimulus, response to stress, immune response, nucleotide biosynthetic process, nucleoside phosphate biosynthetic process, immune system process, and deoxyribonucleotide biosynthetic process) and 3 pathways (including thermogenesis, transcriptional misregulation in cancer, and Parkinson disease) were significantly enriched with down-regulated DEGs at 12 hpi. Similarly, only 2 GO terms (defense response and modulation of synaptic transmission) and KEGG pathways (transcriptional misregulation in cancer and regulation of lipolysis in adipocytes) were significantly enriched in the infection group of 24 hpi. In addition, the biological process or pathways of defense response and transcriptional misregulation in cancer were significantly enriched both in two infection groups. Notably, there were more GO terms and KEGG pathways enriched by down-regulated DEGs in the group of 12 hpi than in 24 hpi.

### The Validation of RNA-Seq

**Figure 3:**
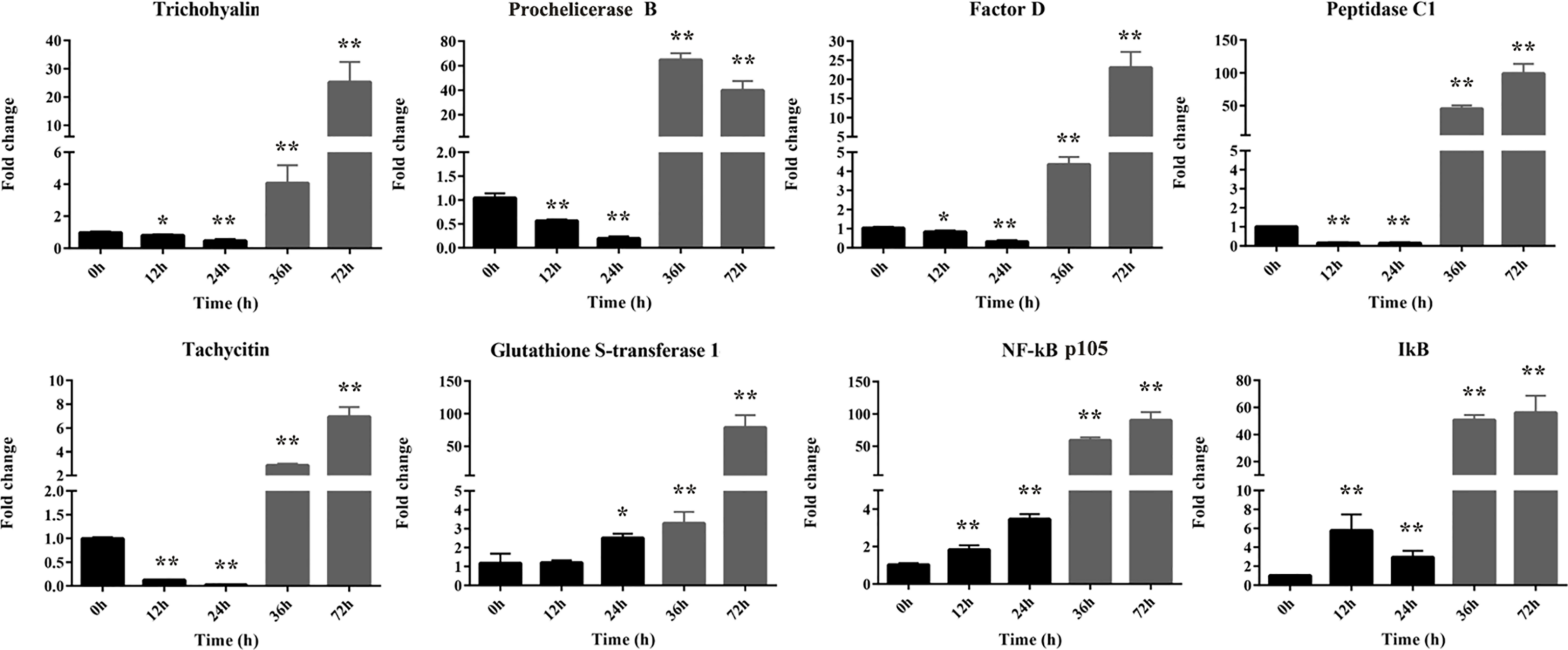
The relative expression levels of eight selected DEGs identified by qRT-PCR. These genes including five down-regulated genes (trichohyalin, Factor B, Factor D, peptidase C1, and tachycitin) and three up-regulated genes (glutathione S-transferase 1, NF-κB p105, and IκB). 18S rRNA was used as the internal reference. Each experiment was executed in triplicate. Data are shown as mean ± SD (N = 3). The asterisk indicates significant difference (**P < 0.01, *P < 0.05) compared with the control (set as 1).

Transcriptional regulation by RNA-Seq data was confirmed in a biologically independent experiment using qRT-PCR. A total of eight genes were selected for qRT-PCR analysis at different time (0∼72 h) after injection. These DEGs included three up-regulated genes (glutathione S-transferase 1, NF-κB [p105], and IκB) and five down-regulated genes (peptidase C1, tachycitin, factor D, Prochelicerase B, and trichohyalin). As expected, NF-κB signaling pathway (including NF-κB and IκB) was activated by Gram-negative bacteria at all time points post infection. On the contrary, the antimicrobial substances of tachycitin and factor D and the prochelicerase B were suppressed at 12 and 24 hpi with Gram-negative bacteria observed in **Figure 3**, although they were significantly highly expressed at 36 and 72 hpi. In addition, three genes from DEPs (including glutathione S-transferase 1, peptidase C1, and trichohyalin) were also identified at the mRNA level. Among them, the glutathione S-transferase 1 gene showed a continuously increasing trend after 24 h of infection. The other two genes, peptidase C1 and trichohyalin had similar changes at all time points, which were suppressed at 12 and 24 hpi but activated at 36 and 72 hpi. The expression patterns of these genes at different time points were consistent with the corresponding transcriptome expression levels. Notably, all of these genes were significantly highly expressed at 36 and 72 hpi compared with the control regardless of their expression levels at 12 and 24 hpi.

### Peptidome Analysis

**Figure 4:**
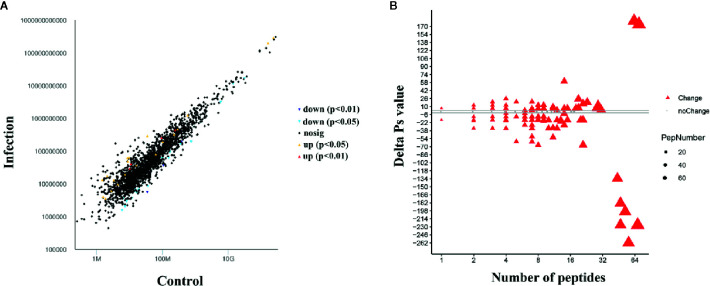
Differentially abundant peptides (DAPs) and differentially expressed protein (DEP) analysis at 24 hpi with Gram-negative bacteria. **(A)** Visualization of differential abundant peptides at 24 hpi. Down and up indicate down-regulated expression and up-regulated expression, respectively. *P* < 0.05 and *P* < 0.01 represent significant differences and extremely significant differences, respectively. **(B)** Quantitative ΔPs analysis between the infection group and control group. Red triangles indicate differential expression, and gray dots indicate no significant difference. The threshold is |ΔPvalue| > 2.

A comparative peptidome survey between the infection and control groups was performed using the label-free technique to complement the transcriptome experiments. A total of 443 differentially abundant peptides (DAPs, shown in **Figure S1**) were identified at 24 h after infection (*P*-value < 0.05). Compared to the control group, 24 DAPs had higher abundance, and 34 DAPs had lower abundance in the infection group (**Figure 4A**). And 170 peptides were expressed only in the infection group, 215 peptides were expressed only in the control group. Through mapping these DAPs to the transcriptome datasets, 208 differentially expressed proteins (DEPs) were obtained. Among these DEPs, 80 proteins were up-regulated and 128 proteins were down-regulated after infection with Gram-negative bacteria (**Figure 4B**).

**Figure 5:**
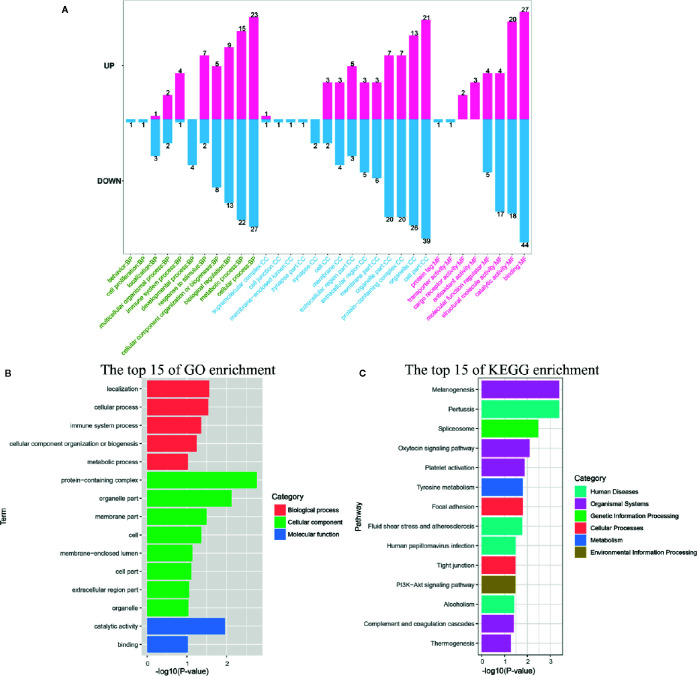
Functional annotation and enrichment analysis of differentially expressed proteins (DEPs). **(A)** The GO classification of DEPs was performed via GO annotation. BP, biological process; CC, cellular component; MF, molecular function. GO **(B)**, and KEGG **(C)** enrichment analysis of DEPs in the infection group.

All DEPs were annotated into 33 terms by GO annotation analysis (**Figure 5A**). Classification of GO revealed that both the up-regulated and down-regulated proteins yielded proteins with similar function involved with biological processes (BP), molecular function (MF), and cell components (CC). The top-three most active GO terms (with the most DEPs) were binding (MF), cell part (CC), and cellular process (BP). Immune-related GO terms including immune system process, response to stimulus, and antioxidant activity were almost enriched with up-regulated proteins. On the contrary, localization, developmental process, and synapse were almost enriched with down-regulated proteins. It was notable that some cytoskeleton formation related GO terms, such as synapse, synapse part and structural molecule activity, were enriched with DEPs in this study. GO enrichment analysis showed that eight GO terms, including protein-containing complex, organelle part, catalytic activity, localization, cellular process, membrane part, immune system process, and cell, were significantly enriched (*P* < 0.05, **Figure 5B**).

Among the DEPs, 103 proteins could be annotated to 164 KEGG pathways. The 13 pathways, including melanogenesis, pertussis, spliceosome, oxytocin signaling pathway, platelet activation, tyrosine metabolism, focal adhesion, fluid shear stress, and atherosclerosis, human papillomavirus infection, tight junction, PI3K-Akt signaling pathway, alcoholism, and complement and coagulation cascades, were significantly different (*P* < 0.05) in response to *V. parahaemolyticus* infection if judged by the *P*-value on the basis of Fisher’s exact test (**Figure 5C**).

### Correlation Analysis of RNA and Protein Expression

To demonstrate the correlation of expression levels in protein and mRNA, all of the proteins obtained in horseshoe crab hemolymph proteome analysis were compared with the mRNAs corresponding to transcriptome analysis. All of the 550 proteins could be matched to transcripts according to their amino acid sequences. However, there was a low Pearson correlation coefficient (r = 0.13, *P* < 0.01) between the changes in the transcription level and protein level for all genes present in both the transcriptomic and peptidome data. A low correlation coefficient was also observed in other studies (33). The reasons for the low correlation may be contributed by different degradation rates of mRNA and protein, post-transcriptional processing, translation efficiency and post-translation modification (34).

A total of 40 genes were identified from both the DEGs and the corresponding DEPs. Among them, 23 genes had the same regulatory direction between infection and control groups at the mRNA and protein levels, including 8 up-regulated genes and 15 down-regulated genes (**Table S3**).

## Discussion

Chinese horseshoe crab is an ancient marine arthropod, which depends entirely on the innate immunity to resist pathogens. To explore the response mechanism to Gram-negative bacteria in Chinese horseshoe crab, expression profiles of the genes in response to *V. parahaemolyticus* infection were analyzed using transcriptomics combined with peptidomics, and the patterns of DEGs (such as NF-κB, IκB, and Prochelicerase B) in defense against *V. parahaemolyticus* have been validated. These results will help us to further investigate the response mechanisms to Gram-negative bacteria in the horseshoe crab hemolymph.

**Figure 6:**
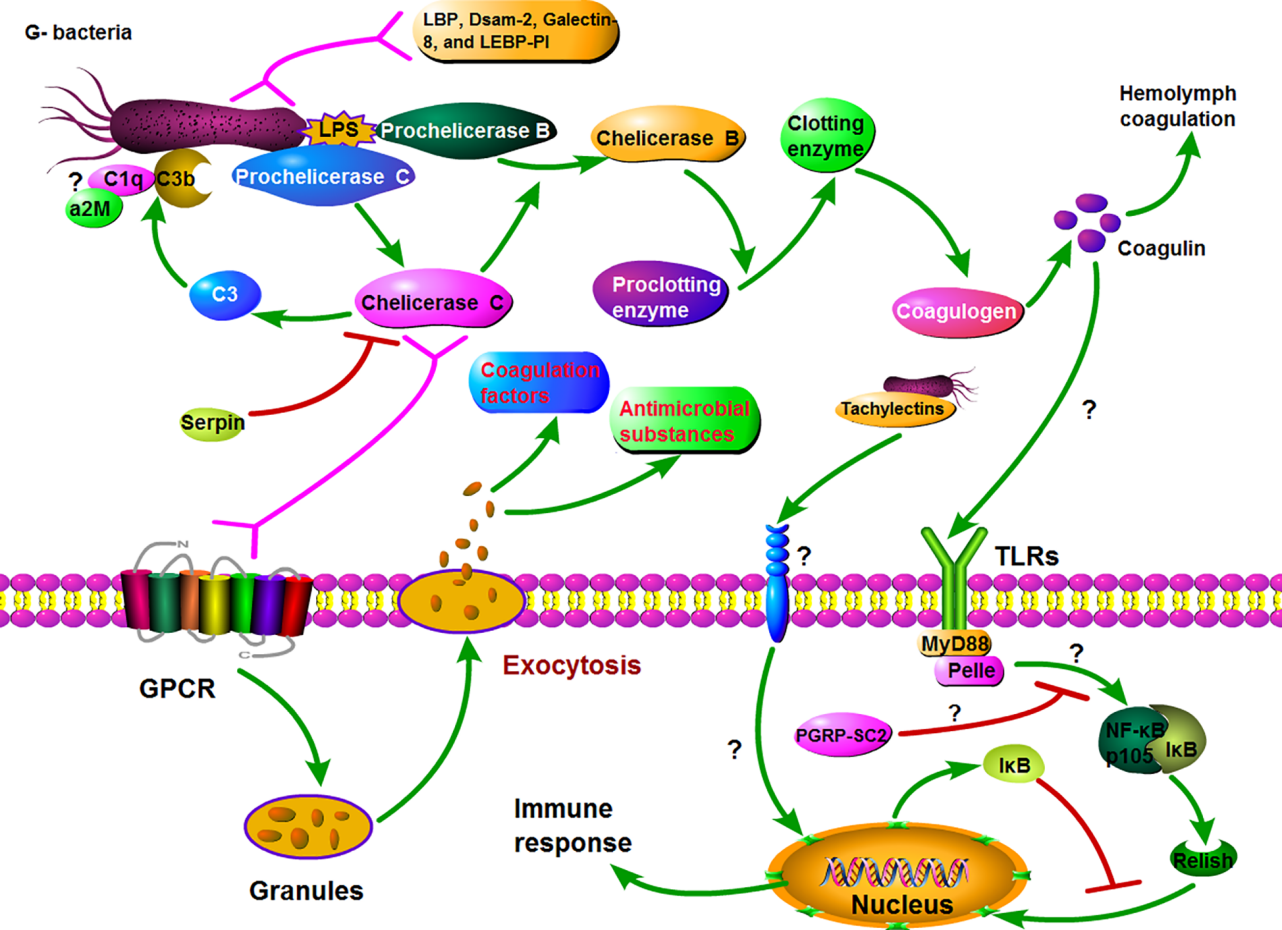
Hypothetical model for Gram-negative bacteria induced major innate immune response in horseshoe crabs. Firstly, Prochelicerase C is induced by LPS and autocatalytically activated on the surface of the bacteria, *whose* active form Chelicerase C activates Prochelicerase *B and then triggers hemolymph coagulation cascades via activating proclotting enzyme to clotting enzyme.* On the other hand, the exocytosis occurs after binding of Chelicerase C with granules hemocyte and subsequent signaling via a G protein-coupled receptor signaling pathway, resulting in the release of various defense molecules, including antimicrobial peptides and coagulation factors. Simultaneously, the coagulin activates the Toll\NF-κB signaling cascade through interacting with tToll, which also leads to the release of antibacterial substances and other immune response.

In the present study, RNA-seq and comparative analysis were performed in the hemolymph at two time points after infection with *V. parahaemolyticus*. According to previous studies, the mRNA expression levels of many key factors (including Factor C, NF-κB and IκB) involved in horseshoe crab *Carcinoscorpius rotundicauda* immune response reached its highest levels between 12 and 24 h after infection with bacteria (8, 35). Therefore, samples at the time points of 12 and 24 h were used for transcriptomic analysis in this study. In addition, the results of transcriptomic analysis showed that there was no significant difference in the number of differentially expressed genes at 12 h and 24 h after infection, and the results of annotation analysis with DEGs were also similar. Therefore, we decided to use the samples at the time point of 24 h for proteomics analysis. As expected, in addition to factors involved with general biological processes, hemolymph expressed a suite of innate immunity genes under the infection state (**Figures 2** and **5**). These genes have previously been shown to be important in the immune defense (**Table 3**), but the cellular localization and function of many of these genes have yet not been characterized in detail, here we discussed below the putative function of some of these innate immunity effectors in the context of Chinese horseshoe crab hemolymph (**Figure 6**). KEGG pathway analysis showed that many pathways including Toll-like receptor, NF-κB, C-type lectin receptor, JAK-STAT, and MAPK signaling pathways were immune response related biologic pathways to *V. parahaemolyticus*. The functions of some genes from these pathways were speculated in other studies (**Table 3** and **Figure 6**).

**Table 3:** Innate immunity-related DEGs from *Tachypleus tridentatus* hemolymph after infection with *V. parahaemolyticus*.

Putative ID	Accession no.	Organism	Putative function	e-Value	Regulation	Protein
NF-κB signaling pathway					12 hpi/24 hpi	24 hpi
NF-κB p105	XP_013780935.1	*L. polyphemus*	NF-κB signaling	7.5E-95	Up/Up	ND
IκB	AAZ40334.1	*C. rotundicauda*	Suppressor of NF-κB	7.3E-217	Up/Up	ND
Relish	ABC75034.1	*C. rotundicauda*	NF-κB signaling	0	Up/NS	ND
Toll signaling pathway						
Protein toll-like	XP_022235721.1	*L. polyphemus*	Toll signaling	0	Up/Up	ND
Toll-like receptor 3	XP_013793330.1	*L. polyphemus*	Toll signaling	1.4E-150	Up/Up	ND
MyD88	XP_013776308.1	*L. polyphemus*	Toll signaling	1.1E-214	Up/Up	ND
Pelle	XP_013790057.1	*L. polyphemus*	Toll signaling	0	Up/Up	ND
Complement and coagulation cascades						
Tachylectin-2	Q27084.1	*T. tridentatus*	Glycan binding	3.8E-34	Down/NS	Down
Tachylectin-4	BAF76636.1	*T. tridentatus*	Glycan binding	1.2E-9	Down/Down	ND
Tachylectin-5A	Q9U8W8.1	*T. tridentatus*	Glycan binding	1.5E-61	NS/NS	Up
Tachylectin-5B	XP_013784867.1	*T. tridentatus*	Glycan binding	2.2E-47	NS/NS	ND
Prochelicerase B	Q27081.1	*T. tridentatus*	Serine protease	1.9E-237	Up/NS	NS
Prochelicerase C	AAL75577.1	*T. tridentatus*	Serine protease	5.3E-26	Up/NS	Down
Proclotting enzyme	XP_013773969.1	*L. polyphemus*	Serine protease	1.3E-184	Up/NS	ND
Complement C3	BAH02276.1	*T. tridentatus*	Complement	0	NS/NS	Up
Complement C1q	XP_013790852.1	*L. polyphemus*	Complement	2.4E-142	NS/Up	ND
α-2-macroglobulin	BAA19844.1	*L. polyphemus*	Complement-like	1.1E-21	Up/Up	ND
Coagulogen	XP_022252435.1	*L. polyphemus*	Gelation	2.1E-46	NS/Up	Up
Serpin	BAA03374.1	*T. tridentatus*	Serpin	3E-224	Up/Up	ND
Antimicrobial substances						
Factor D	BAA13312.1	*L. polyphemus*	Serprocidin	2.7E-160	Up/Up	Down
Big defensin	P80957.2	*T. tridentatus*	Antimicrobial	1.9E-32	Down/NS	ND
Tachyplesin-1	P14213.2	*T. tridentatus*	Antimicrobial	1.9E-30	Down/Down	NS
Tachystatin-A2	Q9U8X3.1	*T. tridentatus*	Antimicrobial	2.4E-33	NS/Down	NS
Tachystatin-B1	P0C1Z8.1	*T. tridentatus*	Antimicrobial	7.5E-18	Down/Down	NS
Tachycitin	BAA12864.1	*T. tridentatus*	Antimicrobial	4.7E-47	Down/Down	Down
Others						
GPCR	XP_013780883.1	*L. polyphemus*	Signaling	8.6E-188	Up/Down	ND
PGRP-SC2	XP_013792255.1	*L. polyphemus*	Peptidoglycan binding	1.5E-27	Down/Down	NS
LBP	AAF74774.1	*T. tridentatus*	LPS-binding	5.2E-61	Down/Down	ND
LEBP-PI	AAA28271.1	*L. polyphemus*	Trypsin Inhibitor	1.4E-39	Down/NS	NS
Galectin-8	XP_013777140.1	*L. polyphemus*	Glycan binding	5.4E-171	Up/NS	ND
Dscam2	XP_022240087.1	*L. polyphemus*	Glycan binding	0	Down/Down	ND

Prochelicerase B, Clotting factor B; Prochelicerase C, Clotting factor C; ND, not determined; NS, not significant; Up, up-regulation; Down, down-regulation.

In proteome data, GO analysis of all 208 DEPs showed that many proteins related to cytoskeletal structure were annotated, which may be due to the rapid deformation of granulosa cells and exocytosis under pathogenic stimulation (**Figure 5A**). GO enrichment analysis showed that biological processes such as protein complexes and catalytic activity were also activated when immune response (GO:0002376 immune system process) was activated by bacteria. KEGG pathways of melanogenesis and complement and coagulation cascades were significantly enriched by DEPs, suggesting that the melanogenesis and hemolymph coagulation probably were major defense mechanism after infection with Gram-negative bacteria in humoral immunity in horseshoe crab.

The first and most important step of the defense mechanism is the recognition of non-self materials. Among those DEGs involved with innate immunity, several putative PRRs were found, including galectin-8, peptidoglycan-recognition protein SC2 (PGRP-SC2), down syndrome cell adhesion molecule 2 (Dscam-2), Tachylectins, LPS-binding protein (LBP), and some proteins (such as Prochelicerase C, Prochelicerase B, and Prochelicerase G) with polysaccharide recognition function. In invertebrates, Galectins, Dscams, Lectins, LBS-binding protein and PGRP-SC2 can bind carbohydrates or lipid and have been implicated as PRRs during the immune response (36–39). In previous studies, Tachylectins have been shown to participate in the recognition of non-self substances including Gram-negative and -positive bacteria and play a key role in the immune defense in horseshoe crabs (40, 41). Tachylectins recognize and agglutinate pathogens *via* the acetyl group on the surface of the microorganism in horseshoe crab (42). In invertebrates, PGRP-SC2 plays important roles in resisting the invasion of bacteria, triggering phagocytosis, activating prophenoloxidase system (43, 44), and suppressing the Imd/Relish signaling in immune response as a negative regulator.

In DEGs, Prochelicerase C, Prochelicerase B, and Proclotting enzyme are a class of key defense factors against invading bacteria. These factors produce a series of enzyme-catalyzed reactions *via* recognizing pathogens, and then activate the coagulation cascade as shown in **Figure 6**. On the other hand, activated Chelicerase C transmits signals *via* GTP binding protein-mediating signaling pathway to horseshoe crab granular hemocytes, causing exocytosis, and releasing antibacterial substances such as antibacterial peptides and lectins (**Figure 6**). It is notable that Chelicerase C can simultaneously activate humoral and cellular immune responses in the horseshoe crab hemolymph.

Toll signaling pathway is considered to be a crucial biological pathway in the innate immune response in *Drosophila*, which involves the process of eliminating invading pathogens, including Gram-negative and -positive bacteria (45). In this pathway, the *Drosophila* Toll (dToll) protein is not a pattern recognition receptor, but plays a key role in signal transmission as a membrane protein. Similar to dToll, the Toll-like protein (named tToll) from Chinese horseshoe crab also has no ability to recognize pathogens. In *Drosophila*, the PGRPs transmit signals to the adaptor protein Sptzle (SPZ) after infection, and the activated SPZ transmits the signal to the hemolymph cell *via* binding with dToll. In this study, two Toll-like proteins (Toll-like protein, Toll-like protein 3) and two key genes (MyD88 and Pelle) in Toll signaling pathway were found in DEGs from the transcriptome datasets. Therefore, we speculate that there is a biological pathway similar to the *Drosophila* Toll signaling pathway in horseshoe crabs. Furthermore, Coagulin is likely the effector that initiates the Toll signaling pathway, because it is considered to be a ligand for TLRs in horseshoe crab (46). It is suggested that the Toll signaling is most likely an important signaling pathway on plasma membrane in immune response in horseshoe crab.

NF-κB signaling pathway is conserved from invertebrates (except *Caenorhabditis elegans*) to vertebrates, playing a pivotal role in regulating the expression of critical immune defense molecules (35). In *Drosophila*, NF-κB signaling is activated by the complex consisting of MyD88, Tube, and Pelle from Toll signaling pathway. The activated NF-κB signaling pathway eventually produces phosphorylated Rel protein, which enters the nucleus to regulate the expression of target genes. Similarly, in horseshoe crabs, the activated Toll signaling pathway may transmit signals to the NF-κB signaling pathway, and induce the Relish protein to enter the nucleus to regulate the expression of immune-related target genes. Although the biological functions of these genes remain to be further elucidated, their existence nonetheless provides additional evidence for a functional TLR/NF-κB signaling cascade.

In DEPs, some complement or complement-like factors such as C3, C2/B, C1q, and α2-Macroglobulin (α2M) were identified. The complement system in vertebrates plays an important role in host defense, in which complement component C3 is essential in the opsonization of pathogens. In horseshoe crab, Chelicerase C acts as a C3 convertase, and activated C3b will be deposited on the surface of pathogens including Gram-negative and -positive bacteria and fungi with other molecules (13, 14). Similar to C3b, complement Factor C1q is also deposited on the surface of pathogens during immune defense (47), but there is no evidence that it has binding activity with Gram-negative bacteria. The α2M is a complement homologue in horseshoe crabs, and its N-terminal structure has a certain similarity with human complement Factor C8γ chain (48). Human C8 is an integral member of the membrane-attack complex. Based on this, we speculate that C3b, C1q, and α2M may form molecular polymers on the surface of pathogens similar to membrane-attack complex. It is also possible that these molecules independently bind to pathogens and play a role in exposing and releasing pathogenic signals.

Among other genes, serpin, the intracellular coagulation inhibitor, has been shown to be a Prochelicerase C inhibitor in the immune defense of horseshoe crabs, which negatively regulates the hemolymph coagulation cascade (1, 49, 50). In addition, although both LBP and *Limulus* endotoxin-binding protein protease inhibitor (LEBP-PI) have LPS binding activity, the biological processes that they participate in are unknown (1). The biological pathways such as C-type lectin receptor, JAK-STAT, MAPK, and melanogenesis are significantly enriched by DEGs or DEPs, which play important roles in immune response in horseshoe crab in our hypothesis though additional evidences will be required.

In conclusion, we used transcriptomic and peptidomic to systematically analyze the innate immune response to Gram-negative bacteria in hemolymph of the horseshoe crab. Our results showed that Gram-negative bacteria could activate the hemolymph coagulation cascade and antibacterial substances release. In addition to some known proteases involved in these reactions, we found that Toll, NF-κB, C-type lectin receptor, IL-17, JAK-STAT, MAPK, melanogenesis, and complement and coagulation cascades pathways were also most likely to participate in the immune response to Gram-negative bacteria in hemolymph. The effectors of C3b, C1q, and α2M may form molecular polymers on the surface of pathogens similar to membrane-attack complex. Moreover, the Toll and NF-κB signaling pathways may constituted an integral signal cascade in immune defense system of horseshoe crab. These results will provide a useful reference for further understanding innate immune response mechanism in horseshoe crabs.

## Data Availability Statement 

The data presented in the study are deposited in the NCBI Short Read Archive (SRA) repository, accession number SRP267502.

## Ethics Statement

This animal study was reviewed and approved by Huazhong Agriculture University Sciences Animal Care Committee.

## Author Contributions

All authors contributed to the article and approved the submitted version. W-FW designed the experiments, analyzed the experiments data, and wrote the manuscript. X-YX, KC, X-LC, and W-LZ performed, analyzed, and interpreted the study. H-LW designed the experiments and revised the manuscript.

## Funding

This work was supported by Science and Technology Major Project of Guangxi (NO. AA17204088), Fundamental Research Funds for the Central Universities (2662019PY036).

## Conflict of Interest

The authors declare that the research was conducted in the absence of any commercial or financial relationships that could be construed as a potential conflict of interest.

## Supplementary Material

Supplementary Figure 1 The DAPs expression patterns between the infection and control groups. Cluster analysis was conducted by the method of hcluster, and the statistical methods for inter-sample and inter-peptide were Spearman and Pearson, respectively.

## Abbreviations

*Tachypleus tridentatus*, *T. tridentatus*; lipopolysaccharides, LPS; pattern-recognition receptors, PRRs; *Tachypleus tridentatus* Toll-like protein, tToll; *Vibrio parahaemolyticus*, *V. parahaemolyticus*; Short Read Archive, SRA; non-redundant, NR; Gene ontology, GO; Kyoto Encyclopedia of Genes and Genomes, KEGG; differentially expressed genes, DEGs; fragments per kilobase of exon per million mapped reads, FRKM; quantitative real-time polymerase chain reaction, qRT-PCR; Standard deviation, SD; false discovery rate, FDR; differentially abundant peptides, DAPs; differentially expressed proteins, DEPs; biological processes, BP; molecular function, MF; cell components, CC; peptidoglycan-recognition protein SC2, PGRP-SC2; down syndrome cell adhesion molecule, Dscam; Tachylectins, TLs; *Drosophila* Toll, dToll; complement component C3, C3; complement Factor C1q, C1q; α2-Macroglobulin, α2M; LPS-binding protein, LBP; *Limulus* endotoxin-binding protein protease inhibitor, LEBP-PI.
