# Supplementary figures and images for: Immune Responses to Gram-Negative Bacteria in Hemolymph of the Chinese Horseshoe Crab, Tachypleus tridentatus
Source: Front Immunol. 2021 Jan 29;11:584808. doi: 10.3389/fimmu.2020.584808 (PMC7878551; doi:10.3389/fimmu.2020.584808)

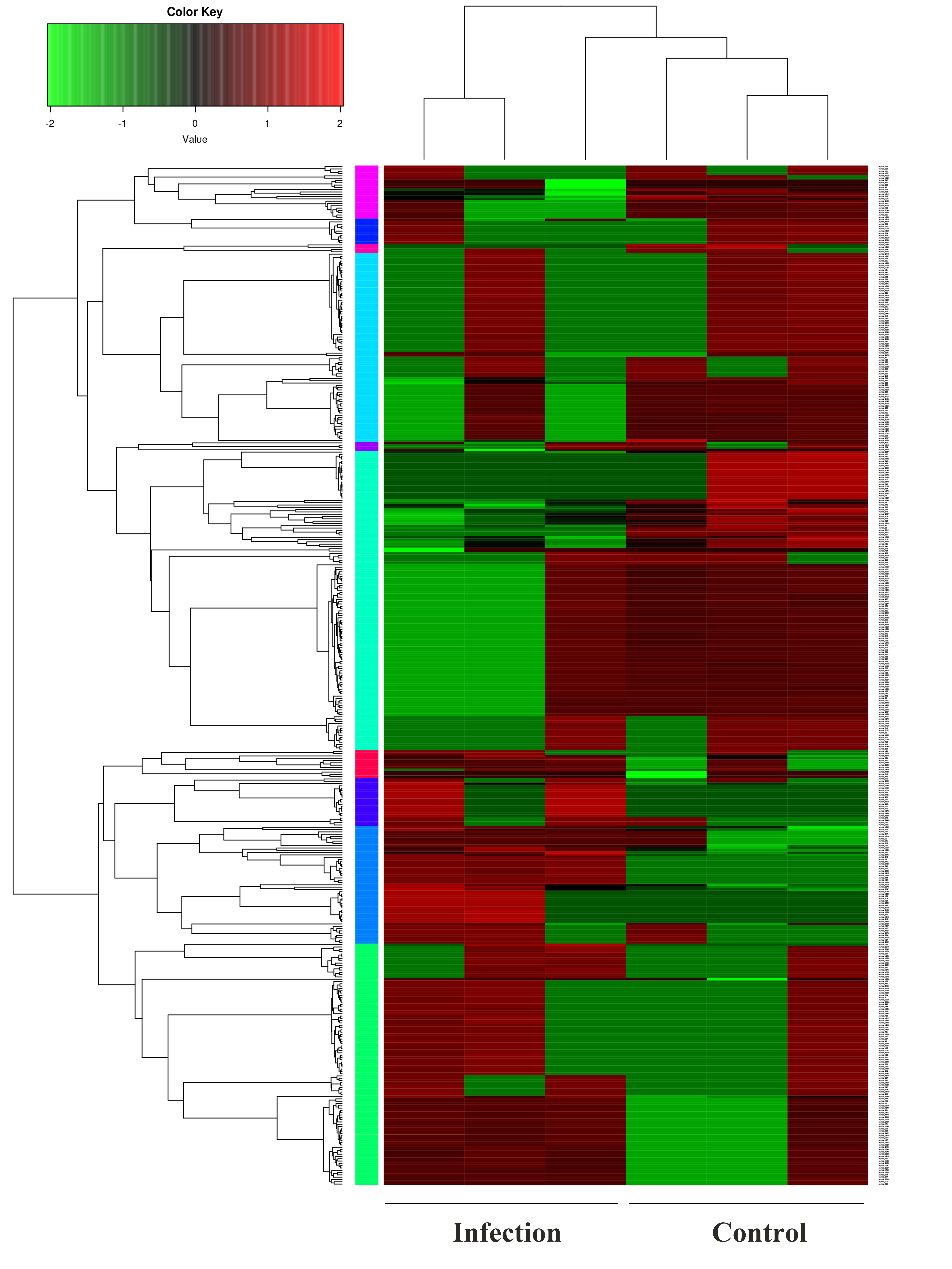

Supplement: Supplementary Figure 1 — The DAPs expression patterns between the infection and control groups. Cluster analysis was conducted by the method of hcluster, and the statistical methods for inter-sample and inter-peptide were Spearman and Pearson, respectively. [file Image_1.tif]
